# 2645. Severe Human Parainfluenza Virus-associated Pneumonia in Adults, Seoul, South Korea, 2010-2019

**DOI:** 10.1093/ofid/ofad500.2257

**Published:** 2023-11-27

**Authors:** Joung Ha Park, Sang-Bum Hong, Jin Won Huh, Jiwon Jung, Min Jae Kim, Yong Pil Chong, Heungsup Sung, Kyung Hyun Doh, Sung-Han Kim, Sang-Oh Lee, Yang Soo Kim, Chae-Man Lim, Younsuck Koh, Sang-Ho Choi

**Affiliations:** Chung-Ang University Hospital, and Chung-Ang Medical Health Care System Hyundae Hospital, Seoul, Seoul-t'ukpyolsi, Republic of Korea; Asan Medical Center, Seoul, Seoul-t'ukpyolsi, Republic of Korea; Asan Medical Center, Seoul, Seoul-t'ukpyolsi, Republic of Korea; Asan Medical Center, Seoul, Seoul-t'ukpyolsi, Republic of Korea; Asan Medical Center, Seoul, Seoul-t'ukpyolsi, Republic of Korea; Asan Medical Center, Seoul, Seoul-t'ukpyolsi, Republic of Korea; Asan Medical Center, Seoul, Seoul-t'ukpyolsi, Republic of Korea; Asan Medical Center, Seoul, Seoul-t'ukpyolsi, Republic of Korea; Asan medical center, Seoul, Seoul-t'ukpyolsi, Republic of Korea; Asan Medical Center, Seoul, Seoul-t'ukpyolsi, Republic of Korea; Asan Medical Center, Seoul, Seoul-t'ukpyolsi, Republic of Korea; Asan Medical Center, Seoul, Seoul-t'ukpyolsi, Republic of Korea; Asan Medical Center, Seoul, Seoul-t'ukpyolsi, Republic of Korea; Asan Medical Center, Seoul, Seoul-t'ukpyolsi, Republic of Korea

## Abstract

**Background:**

Human parainfluenza virus (HPIV) is a major cause of acute respiratory tract infections such as croup in children and pneumonia in immunocompromised patients. HPIV infections are generally regarded as mild and self-limiting in adults, however, HPIV-associated pneumonia is increasingly reported in adults. To the best of our knowledge, data on the epidemiological and clinical characteristics of severe HPIV-associated pneumonia in adults are limited. Therefore, we investigated the epidemiological and clinical characteristics and outcomes of severe HPIV-associated pneumonia over ten years in a large cohort of critically ill adults with severe pneumonia requiring intensive care.

**Methods:**

This sutdy was performed as a part of a prospective observational cohort study regarding severe virus-associated pneumonia at the 28-bed medical intensive care unit (ICU) at a 2,700-bed hospital, in Seoul, South Korea. Patients with severe HPIV-associated pneumonia were enrolled in this study, and the clinical data of these patients were analyzed. To describe the clinical outcomes of severe HPIV-associated pneumonia, we compared the HPIV-associated pneumonia outcomes with those of severe influenza virus-associated pneumonia.

**Results:**

A total of 143 adult patients with severe HPIV-associated pneumonia were identified. HPIV is the most common cause (25.2%) of severe viral hospital-acquired pneumonia (HAP), and it is the third most common cause (15.7%) of severe viral community-acquired pneumonia. The median age of the patients was 61.6 years old. Almost patients (97.2%) had comorbidities, and hematologic malignancy (35.0%), diabetes mellitus (23.8%), and structural lung disease (21.0%) were common cormobidities. About half of the patients (54.5%) had co-infections at the time of ICU admission. The 90-day mortality was comparable with that of severe influenza virus-associated pneumonia (55.2% vs 48.4%, p = 0.22). The use of ribavirin was not associated with lower mortality. Fungal co-infections were associated with higher mortality (82.4% [14/17]).

**Figure:**
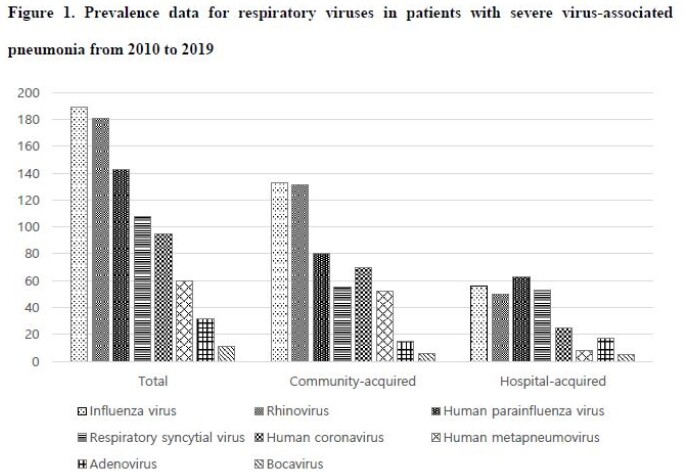

**Figure:**
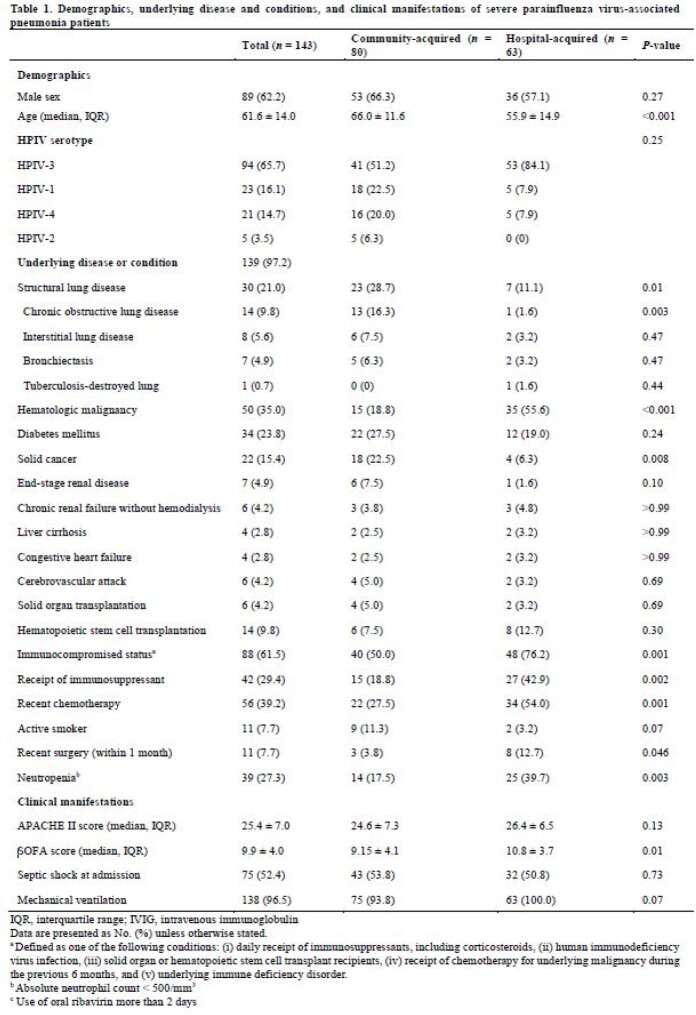

**Conclusion:**

In conclusion, HPIV was the leading cause of severe viral HAP with substantial mortality, comparable to that of influenza virus-associated penumonia. Fungal co-infections contributed to the high mortality rates.

**Disclosures:**

**All Authors**: No reported disclosures

